# Community mobilization and maternal Care of Women Living with HIV in poor settings: the case of Mfuwe, Zambia

**DOI:** 10.1186/s12913-018-2959-3

**Published:** 2018-03-02

**Authors:** Choolwe Muzyamba, Wim Groot, Sonila Tomini, Milena Pavlova

**Affiliations:** 1 Maastricht Graduate School of Governance/UNU-Merit, Maastricht University, Maastricht, The Netherlands; 2 Department of Health Services Research, CAPHRI, Maastricht University Medical Center, Faculty of Health, Medicine and Life Sciences, Maastricht University, PO Box 616, 6200 MD Maastricht, The Netherlands; 3 UNU MERIT, Boschstraat, 246211 AX Maastricht, The Netherlands

**Keywords:** Community mobilization, HIV, Maternal health, Resource-poor settings

## Abstract

**Background:**

Research has shown that community mobilization is a useful strategy in promoting maternal care of HIV negative women in resource poor settings; however, similar evidence for women living with HIV is missing. Therefore, in this study we provide this evidence by exploring the relevance of community mobilization in the promotion of maternal health care among women living with HIV in resource-poor settings by using Mfuwe, a rural district in Zambia as a case study.

**Methods:**

By relying on Focus Group Discussions (FGDs), qualitative data were collected from Mfuwe, Zambia. The data were digitally recorded, transcribed and later translated from CheChewa (local language) to English. We relied on Thematic analysis to analyze the data.

**Results:**

By focusing on community mobilization, our results showed that within their social fabrics, resource-poor communities often contain unrecognized and sometimes ignored strategies which are contextually-feasible and have been used for generations to promote maternal care for HIV positive women. Further, it was evident that although the three forms of community mobilization were largely and uniquely useful in promoting maternal health care of women living with HIV, they also presented unique and various shortcomings.

**Conclusion:**

We demonstrated that community mobilization was largely and often characterized as a force for good (e.g. providing support, improving access to maternal care etc.) and sometimes for bad (e.g. reinforced harmful misconceptions, superstition and stigma). Thus we recommend that community mobilization needs to be factored into maternal health care policies for HIV positive women in resource poor settings either to optimize their potential benefits or to minimize their potential harm.

## Background

In resource-poor settings where health systems are inefficient, research has shown that community mobilization can be a useful strategy for improving maternal care of women who test negative for Human Immunodeficiency Virus (HIV) [1, 2]. Similar evidence for HIV positive women, however, is still lacking despite them being a more vulnerable given their HIV status [1–3]. Community mobilization is defined as any maternal health promotion strategy which makes use of indigenous resources, peer support, and/or actively involves community members in designing and implementing maternal health initiatives. Specifically, community mobilization in this paper refers to one or more of the following three principles: **a) peer support** e.g. support from peers in the form of financial, psychological, social support; **b) utilization of indigenous resources** e.g. trained Traditional Birth Attendants (TBAs); **c) community involvement** which is seen through collaborative partnerships between health professionals and communities in designing and implementing maternal health initiatives [4–6]*.* Aside from the lack of evidence on the effect of community mobilization on maternal care for HIV positive women, there is a lack of evidence on local stakeholders’ (HIV positive women) own perspectives of community mobilization vis-à-vis maternal health care [7, 8]. Such evidence is necessary to design locally suitable and feasible maternal care programs for women living with HIV in resource-poor settings.

Therefore, the aim of this study is to explore the processes and relevance of community mobilization in the promotion of maternal health care among HIV positive women in resource-poor settings using Mfuwe, Zambia as a case study. We explore the local perceptions of community mobilization and how HIV positive women interpret its relevance. For this purpose, we use qualitative data collected in Mfuwe, Zambia. Mfuwe makes a good case study because it is a resource-poor rural area with one of the highest HIV rates and HIV-related maternal deaths in Sub-Saharan Africa [9].

## Theoretical framework

Our study makes use of the social psychological framework known as “community health competence” [1, 10]. The framework is already widely used in HIV research to emphasize the role that informal community group participation plays in HIV response [2, 4, 11]. In this paper, we apply the framework on maternal health of HIV positive women, i.e. the framework guides our analysis and presentation of the results. “Community health competence” is a conceptual framework which enables researchers to assess how health services produce (or fail to produce) health-enhancing environments for local people by relying on “local culture, context and local survival strategies” and collaboration with external experts [12]. This framework emphasizes dialogue and horizontal partnership between indigenous and external efforts in creating health-enhancing opportunities [12, 13]. The framework holds that dialogue between peers, reliance on readily available resources and active collaboration with external experts facilitate the development of heath-enhancing environments more sustainably [13]. This is because the dialogue and collaboration allows vulnerable people (in our case HIV positive women) to critically reflect on their challenges and strengths [11].

The framework indicates that in creating “community maternal health competence” for HIV positive women, the following conditions should be evident: (1) *Peer-support* should be seen to serve as a maternal health-enabler through sharing of maternal health related knowledge among HIV positive women; (2) There should be recognition and use of available “indigenous individuals and social skills” (*indigenous resources*) ([13], p. 5) in responding to maternal health needs of HIV positive women in community; (3) Communities should develop links and work together with external experts in “the public and Non-Governmental Organization (NGO) sectors” to ensure active *community-involvement* in planning and implementation of maternal health initiatives ([13], p. 5).

In this paper, we structure our analysis based on the three conditions espoused in this framework by assessing how peer support, use of indigenous resource and community involvement either promote or hinder maternal health care for HIV positive women in Mfuwe. More specifically, the framework is used to structure the analysis on how community mobilization, specifically its three components (peer support, use of indigenous resources, and community involvement), either facilitates or hinders promotion of maternal care for HIV positive women in Mfuwe, and also highlight the process through which this is achieved.

## Methods

### Setting and target population

Our qualitative study was conducted in February of 2016 in Mfuwe, a rural district in Eastern Zambia. Mfuwe is a rural settlement located in the South Luangwa national park in the Eastern province of Zambia. There exits only one hospital in Mfuwe (Kamoto hospital) catering for a population of over 207,000 people spread roughly around an arear of 370 km^2^. The settlement retains some of the highest HIV rates, including very high maternal and under five mortality rates in the country [14]. It is for this reason that Mfuwe was selected as a case study. Our target population were HIV positive women who had experienced one or more of the components of community mobilization namely: peer support, utilization of indigenous resources, and community involvement.

### Sampling method

In order to select our sample, we relied on purposive sampling technique by establishing contact with two Zambian-based organizations (CIDRZ- Centre for Infectious Disease Research in Zambia and PPAZ- Planned Parenthood Association of Zambia) which were working with HIV positive women in promoting their maternal health care. The two organizations had contact with women who were indirect and direct beneficiaries of the services of these two organizations. Participants were selected with the help of the two local NGOs. Women who met the criteria (were HIV positive and experienced one or more of the components of community mobilization) and expressed interest to participate in the study, were invited to participate. The women all came from different areas/villages within Mfuwe. In order to increase the diversity of opinions expressed by participants and to allow for varied discussions, the diversity of participants was ensured through diversifying the age, educational level, marital status, and employment status of participants.

### Ethical approval

We obtained written ethical clearance from the National Health Research Authority of Zambia. Informed consent in writing was sort from the participants before participation, and at the same time, participants were clearly informed of their right to discontinue their participation in the interview at any point should they wish to.

### Data collection method

Data collection consisted of three Focus Group Discussions (FGDs) all involving 37 HIV positive women who had experienced one or more of the components of community mobilization. The first FGD focused on peer support and had 13 participants, the second one on utilization of indigenous resources had also 13 participants, and the last one on community involvement had 11 participants.

### Guide for interviews

All FGDs lasted between 90 to 100 min. The first author, a Zambian national conducted all the FGDs in Chichewa (the local language) and English was used where possible. Participants were tracked through the discussion using numbers assigned to them (instead of real names) in order to ensure confidentiality and also allow for easy follow up during discussions. A topic guide was used during FGDs but at the same time, discussion of emergent areas of interest to the participants was allowed. In line with the framework presented above, we structured a topic guide that endeavored to elucidate how peer support, use of indigenous resource and community involvement either promote or hinder maternal health care for HIV positive women. This was done through asking questions that allowed participants explain the different ways they experienced the different components of community mobilization, how useful they found the components and what the challenges were. We also ensured that follow-up questions were asked for clarification purposes, and to also encourage respondents to expand on some of their answers. All FGDs were digitally recorded.

### Analysis

We first transcribed the data and then translated them into English, after which, we used the software NVivo 10 QSR International to conduct thematic analysis [15]. By relaying on our theoretical framework, we focused our attention on assessing how peer support, use of indigenous resource and community involvement either promote or hinder maternal health care for HIV positive women in Mfuwe. For this, we identified codes using NVivo. Each specific component of community mobilization gave rise to basic themes that highlighted several and diverse ways in which community mobilization either promoted or hindered maternal care. These basic themes were then systematically clustered to form more structured and elaborate organizing and global themes that show the diverse ways community mobilization prompted and hindered maternal care. After re-reading and refining the themes, we identified 20 organizing themes and 6 global themes (see Table 1).

**Table 1:** Coding frame

Community mobilization component	Global theme	Organizing theme	Basic theme identified in the FGDs
Peer Support	Peer-support as a maternal health-enabler	Supported promoted dialogue	Dialogue helped challenge and re-evaluate inaccurate stereotypes and harmful clichés
			Dialogue enabled sharing information regarding best practices during maternal care
			Dialogue gave rise to empathy and availability of strong friendship ties with people in similar situation
		Provided of support to peers	Peer support promoted emotional, psychological, physical and economic support among HIV positive women during maternity
		Promoted treatment-adherence	Peer support encouraged regular and consistent uptake of ARVs before and after birth
			Peer support served as a continuous reminder for uptake of ARVs
		Fostered alliances	Peer support allows for the formation of alliances among peers to advocate for an end to sexual cleansing
			Working together to fight stigma and discrimination through advocacy and other means
			Fighting patriarchy and promoting women empowerment
	Peer support as a maternal-health inhibitor	Re-enforced superstition regarding institutional-delivery	Peers reinforced the negative superstition regarding health facilities e.g. Clinics practice witchcraft and infant deaths for ritual purposes etc.
		Reinforced a sense of helplessness and dependency	Experiencing and seeing fellow peers’ ill outcomes reinforces helplessness and hopelessness in others
		Promoted harmful sexual practices	Promoting Traditional practice of dry sex
			Promoting Sexual cleansing
Use of indigenous resources	Utilization of indigenous resources as a maternal health-enabler	Trained TBAs provided support	Provide pragmatic services in the form of psychological and emotional support
			Provide adherence-to-treatment support
			Help in providing priority attention to HIV positive women upon recommendation at the facility
			Provide continuous home-based maternal care
		Trained TBAs provided maternal health information	Provide useful maternal health information
		Trained TBAs as a conduit for referrals	Help to refer patients to facilities
			Provide transportation support for women to go to facilities
	Utilization of indigenous resources as a maternal health-inhibitor	TBAs obscured institutional delivery	Presence of TBAs prevents people from seeking professional help
			Presence of TBAs prevents government from improving insertional care
		TBAs lacked skills, equipment and medical supplies to handle complications	TBAs lack skills to Help in PMTCT
			TBAs lack skills to Help Easily conduct HIV tests
			TBAs lack skills to Help in the provision of ARVs
			TBAs cannot Help in conducting caesarian births
community involvement	Community involvement as a maternal health-enabler	Promoted use of Zambulance	Use of Zambulance to transport pregnant mothers to facilities for antenatal, childbirth and postnatal care
			Zambulance help to provide utility transportation services for drugs in difficult terrains
		Promoted use of ‘waiting shelter’ (shelters where expectant mothers can stay while they await delivery.)	Provision of safe spaces for discussing best ways of providing shelter to pregnant women and new mothers
			Provision of care and support to other women within the community through the shelters
			Use of local leaders and other significant people in communities to promote use of shelters
			Provision of nutrition and other supplies necessary during child birth in shelters
			Provision of mosquito nets to prevent HIV positive women against Malaria
			Encourage uptake of institutional delivery among HIV positive women
			Work together with others to encourage other HIV positive mothers seek antenatal and postnatal care
	community involvement as a maternal health-inhibitor	Reinforced tokenism	Community involvement was just symbolic as it failed to actively and realistically involve locals
		Reinforced negative power relations	More powerful NGOs and health workers obscured the voices of the weak and vulnerable

## Results

Our respondents varied in age, marital status and economic activity. Details of the socio-demographic characteristics of our respondents can be found in Table 2. We start by presenting the coding frame after which we give a narrative account of the results. We relied on thematic analysis as shown in Table 1 to assess how the three different components of community mobilization promote or hinder maternal care for HIV positive women in Mfuwe.

**Table 2:** Participants demographics

ID	Age range	Education Level	Employment status
1st FGD (On Peer Support)			
1	20–30	primary education	Employed
2	20–30	Primary education	Employed
3	30–40	No education	Unemployed
4	30–40	No education	Unemployed
5	20–30	No Education	Employed
6	20–30	Primary Education	Employed
7	20–30	No education	Employed
8	20–30	Primary education	Unemployed
9	20–30	Secondary Education	Employed
10	20–30	Primary Education	Unemployed
11	30–40	No Education	Employed
12	30–40	Secondary Education	Employed
13	20–30	Secondary Education	Unemployed
2nd FGD (Utilization of indigenous resources)			
14	30–40	Primary education	Unemployed
15	20–30	Secondary Education	Employed
16	20–30	Primary Education	Unemployed
17	30–40	Primary Education	Employed
18	30–40	No Education	Employed
19	20–30	No Education	Unemployed
20	20–30	Primary Education	Employed
21	20–30	Primary Education	Employed
22	20–30	No Education	Unemployed
23	40–50	Secondary Education	Employed
24	20–30	Vocational training	Employed
25	40–50	No Education	Unemployed
26	30–40	No Education	Unemployed
3rd FGD (Community-Involvement)			
27	20–30	Primary Education	Employed
28	20–30	Secondary Education	Employed
29	40–50	Primary Education	Unemployed
30	30–40	Primary Education	Employed
31	30–40	No Education	Employed
32	30–40	No Education	Unemployed
33	30–40	Primary education	Employed
34	20–30	Secondary Education	Employed
35	20–30	Primary Education	Unemployed
36	30–40	No Education	Unemployed
37	40–50	Secondary Education	Employed
38	30–40	Secondary Education	Employed

### Peer support

In an effort to fight different challenges that HIV positive women faced during maternity, most women in the FGD on peer support refer to peer-support as a safe space for dialogue amongst peers, which enables sharing of helpful maternal health-related information specific for HIV positive women (such as PMTCT- Prevention of Mother To Child Transmission), and challenge misconceptions regarding HIV and pregnancy. The dialogue with peers also enabled these women to take ownership of previously alien HIV treatment and maternal health information. Some of these women also pointed out that the peer-support within the women’s groups in the villages was based on empathy and real friendship, a factor which was necessary to balance their vulnerability as they previously felt disenfranchised and marginalized within their own communities due to their HIV positive status. This is especially emphasized in some quotes from some of the women in the Mfuwe HIV/AIDS women’s’ group:

*“It is easy to see that for a long time, people in this village were stigmatizing and discriminating against us, but now because we speak with one voice, we are able to confront this evil”* Partciapnt:3.

In Mfuwe, professional maternal care facilities were lacking [16], peer support was thus viewed as a useful component of maternal care. Mfuwe is faced with several challenges that include hard-to-reach health centers, poor transport systems, and lack of adequate health workers. According to the majority of the women, challenging these limitations through peer support allowed them to develop agency (the motivation and capacity) to foster improved care for themselves and also to assert their needs in relation to their HIV-vulnerability. This is embodied in the following quote:

*“what is important to us as a group is that we remain supportive to each other through different forms. I have personally benefited emotionally and also physically because my colleagues where present to offer me good nutrition and encourage me to take my medication throughout the process”* Participant 9.

Although peer support was largely characterized as a source of maternal health care support, two participants highlighted some shortcomings associated with peer-support. Specifically, peer-support was said to reinforce misconceptions, superstition and stigma regarding professional maternal care services and sexual reproductive health in general. These respondents pointed out that, through their interaction with other HIV positive women, they developed suspicions regarding condom use, and antiretroviral treatment, which they argued was against their traditional values. They observed that negative maternal outcomes from their peers (fellow HIV positive women) robbed them of confidence and hope for better maternal outcomes for themselves. This resulted in them losing the agency to challenge maternal health obstacles present in their communities.

*“Culture is very important to us, and we are now losing it through these groups. We are adopting foreign ways of doing things and now they are introducing drugs that we don’t know about”* Participant 1.

*“They are now discouraging us from living our sexual lives the way we have been doing over the years. They are busy talking about safe sex and some of my colleagues and I within the group have had to challenge some of these recommendations because they put our marriages at risk. How can a woman tell her husband to use condoms? That is disrespectful to him and against our culture*” Participant 13.

### Use of indigenous resources

Given the limitations in the health system in this rural area, the majority of women in the FGD on the use of indigenous resources intimated that TBAs played a vital role in ensuring quality and affordability of maternal care. Respondents reported that TBAs played a huge role in provision of supportive services to HIV positive women such as social, psychological and emotional support, including referrals to health centers.

*“They (trained TBAs) continue to offer us emotional and psychological support. I remember when I just came out of labor and was brought home; it is Mrs. X (ammonized local trained TBA) our community TBA who took care of me. I was feeling very depressed not knowing whether my child will also become (HIV) positive, but she supported me and encouraged me that all will be well. And I always thank her for that”* Participant 23.

Most respondents praised TBAs for providing useful sexual reproductive health information, encouraging HIV positive women to attend antenatal, childbirth and postnatal care in facilities, as well as supporting HIV positive women to adhere to treatment. This is captioned in quote from some respondents who pointed out that:

*“After she (the TBA) discovered that I was HIV positive, she explained to me the dangers of giving birth at home and as such, encouraged me to start going for antenatal and to give birth at the clinic”* Participant 17.

*“The local TBA always checked on me to make sure that I was taking my drugs. I felt very cared for” *Participant 14.

It was also clear however that a few other respondents were critical of TBAs. Particularly, three of them were concerned about how effective the TBAs were in the community since trained TBAs did not possess necessary skills and medical supplies to conduct caesarian births (a necessary procedure for PMTCT) and handle other complications such as sepsis, hemorrhage etc.

*“it would have been much better if TBAs could operate on me and allow me to give birth using caesarian birth. But they do not have the skills. This is why we go to the clinic. HIV is very problematic and weed to ensure that is perfect care for people like us”* Participant 19.

A few other respondents were also troubled by the governments’ directive to avoid TBAs, including trained TBAs, in preference for clinical care. They stated that although desirable, clinical care was impractical in their case since most clinics were remotely located, overcrowded and lacked essential medication. They also pointed out that the staff in clinics were unfriendly and did not provide quality care.

*“nurses are even worse. They always angrily shout at us and show no sympathy for our situation”* Participant 20.

### Community involvement

During the FGDs, most participants in the FGD on community involvement stated that they specifically collaborated with experts on the following two maternal health initiatives: the “Zambambulance” initiative and the ‘waiting shelter’ project.

The Zambulance (a makeshift ambulance consisting of carriage pulled by a bicycle operated by local people) which is a joint project between the community and the local clinic actively involved local people during planning and implementation. Local drivers volunteered on a rolling basis to transport pregnant women to health facilitates for antenatal, childbirth and postnatal care. Most of the respondents stated that the Zambulance initiative was responsible for increasing access to maternal health services, including access for several HIV positive women who would have otherwise been left unattended to due to infrastructural barriers that existed in Mfuwe.

*“Our clinic doesn’t have an ambulance, and to make matters worse, it is very far from our village. This is why we are using the Zambualnce to transport each other to clinics. Women who are HIV positive have been given priority”* Participant 28.

The creation of “waiting shelters” close to clinics by local people to complement the shortage of bed spaces in the health facility was also hailed for improving access and affordability to maternal care, including care for HIV positive women. Local people while working with experts have set up “waiting shelters” which provide informal support to women (including HIV positive women) in the form of nutrition, psychological and other services necessary for women about to give birth or who recently gave birth. Most respondents praised the manner in which local people and experts collaborated on this idea which proved useful to their community.

*“Unlike what happens in other villages, our village has done well in collaborating with the clinic, nurses and doctors to bring about the waiting shelter. I was there for six days after giving birth and the volunteers were very nice to me”* Participant 29.

Two other participants however criticized these initiatives by branding them tokenistic. They claimed that although collaborative initiatives were well-intended and with great potential, their successful implementation was constrained by the fact that villagers sometimes found themselves on the wrong side of the power-relations. Specifically, powerful NGOs always dictated the direction of the projects while suggestions from locals were often looked down upon. Furthermore, poverty, very few economic opportunities for volunteers and lack of wider political support negatively affected possibilities of scale-up. Respondents asserted that some initiatives (such as Zambulance and waiting shelter) remained symbolic and superficial and did not address the major root-causes of poor maternal care for HIV positive women.

*“It’s good that we have such initiatives but to be honest, the problems in this village are deep-rooted in poverty and lack of jobs for most of the volunteers and us. So what can a poor woman like me say to influence the activities? We have not really addressed the real problems, that is why sometimes you see that we have no food in the shelters and volunteers are fewer these days”* Participant 37.

## Discussion

In this study, we assessed the ways through which community mobilization via its three components (peer-support, utilization of indigenous resources and community-involvement) promote or hinder maternal care of HIV positive women in Mfuwe. Our findings suggest that Mfuwe, a community faced with a health system that has critical material, symbolic and institutional limitations, does have within its social strides several unrecognized and sometimes ignored “portfolios of assets” [2]. These portfolios have over the years formed the cornerstone of maternal health care for women living with HIV [11].

The mainstream narrative has traditionally reflected an embodiment of western-precipitated and ‘universally optimal’ solutions that usually include scaling up of ‘scientific’ and biomedical approaches (while ignoring local strategies and context) as a basis for improving maternal care [17]. This narrative has obscured discussion on what the relevance of community mobilization has been in maternal care of HIV positive women in resource poor settings [2, 18]. In line with the principles of the maternal community health competence framework, our results indicate that building maternal health competence for HIV positive women in local settings requires formal recognition of roles played by experts as well as local actors. This means taking full cognizance of the shortcomings and strengths of both experts and local strategies while paying attention to local context. The results in this paper show that the three components of community mobilization each have strengths and shortcomings in the context of maternal health care of HIV positive women in Mfuwe, Zambia.

### Peer support

For example, peer-support was crucial in providing much needed empathetic social, psychological and emotional supportive services to HIV positive women who in most cases faced stigma due to their HIV status and sometimes suffered the negative consequences of an inefficient health system. Further, by engaging in dialogue with their peers, HIV positive women were able to develop agency and collectively challenge common obstacles to maternal care. At the same time, although peer support generally seems to be a useful maternal health care enabler, it can also act as a negative force. Particularly, it was evident from some respondents that by engaging with peers, several health-inhibiting and harmful sexual and reproductive misconceptions, superstition and stigma were reinforced. This inevitably highlights the challenge that characterizes common practice in public health circles where maternal health initiatives such as peer-support are viewed as a binary (either bad or good). This practice obscures variety [11]. Peer-support, especially if aimed at maternal care of HIV positive women can simultaneously be a force for good and bad. Although for most women the advantages of peer-support seem to outweigh the disadvantages, ignoring its shortcomings would however be counterproductive [2].

### Indigenous resources

Another fact worth noting from our findings is how indigenous resources particularly trained TBAs are characterized in Zambia, how they are used to solve local maternal care challenges and their resulting shortcomings. In the absence of a well-functioning professional health care system, HIV positive women have continue to rely on historically and culturally established local assets such as trained TBAs which have been passed on from generation to generation [17]. Trained TBAs are characterized as useful by HIV positive women in the provision of ‘soft services’ that include treatment-adherence-support, provision of maternal health information, challenging stigma, discrimination and patriarchy, logistical support, nutritional and referral services to facilities. It is also clear that trained TBAs have their limitations reflected in their lack of appropriate skills and resources to tackle pregnancy complications and effectively promote PMTCT. This is why the government of Zambia has outlawed their practice [19]. The action by the government of Zambia (which is influenced by the World Health Organization (WHO) policy on TBAs), however, may worsen rather than improve the condition of the local people of Mfuwe as professional health care is either lacking or riddled with limitations [19]. This is in line with what other studies from different parts of Africa have shown, which is that TBAs form a useful link of care in African societies, thus there is more to benefit from relying on them rather than excluding them from the line of care [20].

### Community involvement

Further, we showed how local people in Zambia characterize and operationalize community involvement in their effort to promote maternal care. Here we established what the community health competency promulgates; which is that local communities often possess useful portfolios which need to be scaled up through horizontal collaboration with experts. Specifically, communities in Mfuwe, Zambia were actively involved and collaborated with external professionals to promote maternal health care of HIV positive women mainly through two distinctive strategies, namely the “Zambambulance” initiative and the ‘waiting shelter’ initiative. This strategy was important in ensuring that HIV positive women who would have otherwise given birth at home were attendant to by professionals in order to promote PMTCT. The two initiatives also had significant local-buy-in and were considered highly useful by the locals. This observation is in consonance with what Renedo & Jovchelovitch (2007) and Campbell and Cornish (2012) in their studies on African communities show which is that by collaborating and “strengthening indigenous responses”, there is increased propensity for HIV positive women to embrace the potential benefits of a given maternal health care strategy. This therefore calls for enhanced recognition on the part of policy makers, NGOs and other stake-holders of the role that partnership plays in promoting enhanced maternal care of HIV positive women. That notwithstanding, in the process of promoting partnership with locals, certain risks seem eminent as expressed by our respondents. There is always the risk of reinforcing tokenism and establishing a base for the struggle of powers between the more-powerful voices (usually experts) and the weaker voices (usually local uneducated villagers). Such events as Campbell and Cornish (2012) observe usually result in situations where more powerful factions invariably dictate the direction of initiatives (in a gaze of partnership) whereas the poor and vulnerable local people are used only as ‘rubberstamps’.

This means that it is imperative to achieve an optimal stage where expert and indigenous factions can mutually dialogue, and “proactively and consciously develop spaces for consensualization” in promoting partnership for purposes of improving maternal care of HIV positive women is not an easy one [3, 21]. This is key in assuring local buy-in, sustainability and success of a maternal health care initiative. Active involvement of locals provides the momentum and agency for local people to define in their own terms what is important to them regarding maternal care and the best way to achieve the goals of maternal care efforts in their given context [22]. This process effectively shades light on what is locally feasible, desirable and the processes which are contextually-suitable to enhance maternal health care for HIV positive women. Jovchelovitch (2012) states that it is folly to ignore local strengths and partnerships, an action which she intimates has become a toxic cornerstone of mainstream maternal health care strategies. Externally-defined local people’s needs and implementation strategies take away their agency to change their situation (despite them possessing indigenous portfolio assets) and discourages ownership of initiatives. This does not however mean that external support is irrelevant; on the contrary, external support is important in several ways (political support, transfer of skills and knowledge etc.). local communities are faced with limitations (as is external support), this is why we contend that in a quest to improve maternal health care of HIV positive women, horizontal collaboration and “strengthening of indigenous responses” rather than obscuring them seems more profitable. This further supports and strengthens the community health competence’s claim that there is more to benefit from building on local strategies through collaboration rather than obscuring them.

### A synthesized view of all the three components

Drawing inferences from the manner in which the three components of community mobilization promote/hinder enhancement of maternal care of HIV positive women, it appears that the change process is neither linear nor replicable across different social settings. It embodies “variety, complexity and fragmentation” ([4], p. 6). Social settings in which HIV positive women reside differ across time and space, and that is why the act of uncritically adopting grand conclusions of maternal health initiatives with the aim of replication seems disingenuous. For example, just because the “Zambulance” initiative works in Zambia does not necessarily mean it will work in other countries as well. This is in line with what Foucault (1980, 1982) postulates that pathways for social transformation across time and space are complex, and failure to take into account this complexity is counterproductive. It is because of this observation that community mobilization’s main principle of promoting “a pastiche of context-specific tactics and homegrown strategies” as a way of promoting maternal care of HIV positive women seems valuable ([4], p. 8). This means that the understanding and application of community mobilization through its three components prioritizes social contexts (which offer different opportunities and challenges for maternal care of HIV positive women) stands in contradiction with mainstream’s characterization of maternal health initiatives as “one-size-fits-all”.

All in all, our findings are in line with what the community health competence framework stands for. Specifically, the stakeholders in our study believe that public health initiatives that genuinely allow for local participation and reliance on indigenous portfolios are likely to promote sustainable access to health care. As suggested by the stakeholders in our study, the core principles of the community health competence produce social spaces for brainstorming on strategies that are practical and feasible by relying on already existing community resources and collaboration with experts. Consequently, through the community health competence framework, our study confirms the benefits of paying attention to diverse ways in which community mobilization hinders and promotes maternal health of HIV positive women in a very context-specific way. This is particularly important for policy as it will allow for scale-up on strategies that promote care for HIV positive women and mitigation for those that hinder maternal care.

### Limitations

We also acknowledge some potential limitations associated with this study. Firstly, our findings are based only on the views of HIV positive women residing in a single province out of the ten provinces in Zambia. This fact limits the variety of experiences with community mobilization in Zambia in general. However, we argue that this study provides useful insights and the first step in understanding the different ways community mobilization hinders/promotes maternal care of HIV positive women in resource-poor settings.

## Conclusion

We set out to assess the ways through which community mobilization via its three components promotes/hinders maternal care of HIV positive women in Mfuwe, Zambia. Given the material, symbolic and institutional limitations that define the health system in Zambia, community mobilization (through its three components) was a dominant feature in maternal health care response of HIV positive women in rural Zambia. Specifically, it was shown that community mobilization was largely and often characterized as a force for good (e.g. providing support, improving access to maternal care etc.) and sometimes for bad (e.g. reinforced harmful misconceptions, superstition and stigma). In line with the community health competence, our evidence suggests that community mobilization needs to be factored into maternal health care policies for HIV positive women in resource poor settings either to optimize their potential benefits or to minimize their potential harm.

Within their social fabrics, resource-poor communities often contain “unrecognized and sometimes ignored portfolio assets” which are contextually-feasible and have been used for generations to promote maternal care for HIV positive women. Ignoring these portfolios or pathologizing local knowledge systems may be self-defeating. It is also worth noting that the different components of community mobilization have different effects on maternal care efforts of HIV positive women in different social settings, which is something that needs to be accounted for. Further, we argue that maternal care change-process for HIV positive women is neither linear nor replicable across time and space. It is characterized by “variety, complexity and fragmentation” ([4], p. 6). This type of change-process consequently questions the legitimacy of common practice in mainstream public health circles to ‘replicate’ and establish “one-size-fits-all” maternal initiatives for HIV positive women based in different settings. This act of romanticizing these uncritical grand-conclusions and ‘replicable’ maternal health strategies is unrewarding in the real world. This is why we argue that embracing the complexity, variety and fragmentation by relaying on community mobilization which prioritizes “a pastiche of context-specific tactics and homegrown strategies” as a way of promoting maternal care of HIV positive women seems valuable ([4], p. 8).

## Abbreviations

ART: Antiretroviral Treatment
CIDRZ: Centre for Infectious Disease Research in Zambia
FGD: Focus Group Discussion
HIV: Human Immunodeficiency Virus
NGO: Non-Governmental Organization
PMTCT: Prevention of Mother to Child Transmission
PPAZ: Planned Parenthood Association of Zambia
TBA: Traditional Birth Attendants
WHO: World health Organization
